# Enhancement in corneal permeability of riboflavin using calcium sequestering compounds

**DOI:** 10.1016/j.ijpharm.2014.06.007

**Published:** 2014-09-10

**Authors:** Peter W.J. Morrison, Vitaliy V. Khutoryanskiy

**Affiliations:** School of Pharmacy, University of Reading, Whiteknights, P.O. Box 224, Reading RG6 6AD, United Kingdom

**Keywords:** Cornea, Epithelium, Calcium chelators, Permeability, Riboflavin, Ocular drug delivery

## Abstract

Ethylenediaminetetraacetic acid, ethylenediamine-*N*,*N*′-disuccinic acid and ethylene glycol-bis(2-aminoethylether)-*N*,*N*,*N*′,*N*′-tetraacetic acid are polyaminocarboxylic acids that are able to sequester metal ions. Calcium is implicated in maintenance of intercellular matrix, zonula occludens (tight junctions) and zonula adherens of epithelium and endothelium cells. Corneal epithelium is impervious to many aqueous formulations due to it being lipophilic, whereby transcellular drug transit is resisted, whilst tight junctions restrict access via the paracellular route. Research has shown that integrity of tight junctions breaks down through loss of Ca^2+^ for endothelial and epithelial cells. This study investigates different Ca^2+^ sequestering compounds and their effect on corneal permeability of riboflavin at physiological pH. Riboflavin is a topically administered ocular drug applied during UV-induced corneal cross-linking for the treatment of keratoconus.

## Graphical abstract

**Figure:**
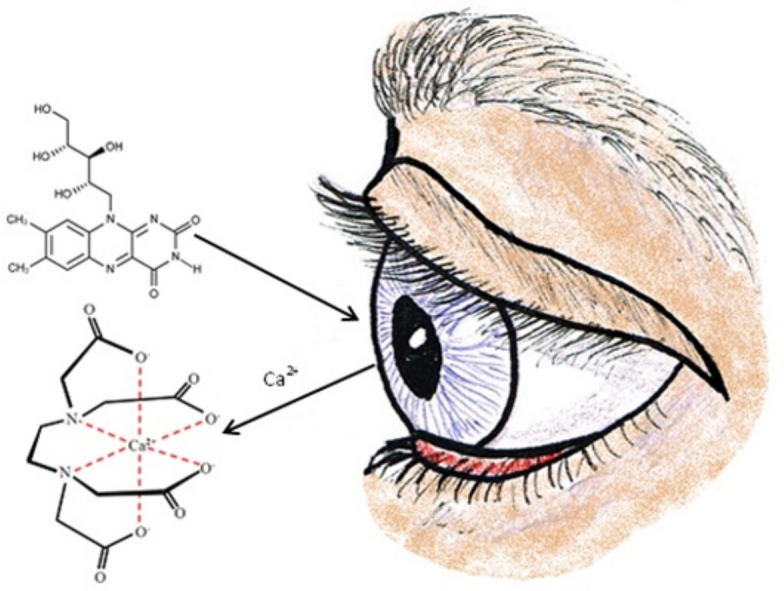

## 1 Introduction

Administering drugs to treat ocular conditions is confronted with many challenges despite easy access to the eye. Drug delivery is hampered by blink action, washout by tears, mechanisms of nasolacrimal drainage and poor permeability of the cornea (Gilhotra et al., 2011; Kumaran et al., 2010). Once the dosage form is applied there is only a short time where the medication is in contact with the eye, during this time drugs intended for intraocular tissue have to penetrate the cornea (Washington et al., 2001). A small proportion of topically applied drugs are absorbed into the cornea, up to 5% but often much less (Jarvinen and Jarvinen, 1996). Most of the instilled dose is systemically absorbed by the nasolacrimal duct and conjunctiva (Wilson et al., 2001). The cornea is a multilayered structure consisting of five layers: epithelium, Bowman’s membrane, stroma, Descemet’s membrane and endothelium. The epithelium is a lipophilic layer of around 10% of the total corneal thickness, offering a barrier of around 90% to hydrophilic drugs and 10% to hydrophobic drugs (Washington et al., 2001; Wilson et al., 2001). The Bowman’s membrane forms a thin transitional layer towards the stroma, which is the main section at ∼90% of the total corneal thickness with hydrophilic gel properties comprised of collagen fibrils, other proteins and mucopolysaccharides. There exists an aqueous environment between collagen fibres, and this contributes to the barrier function that the stroma offers against lipophilic drugs. The next layer is the Descemet’s membrane, a tough but thin, homogenous layer deposited by the endothelium, a single layer of cells important in maintaining optimal corneal hydration (Washington et al., 2001; Wilson et al., 2001). Animal eyes are often used as models when researching ocular drug delivery because they share many of the features present in human eyes. Bovine eyes prove to be recognized models, and these were employed in this investigation (Chandran et al., 2008; Loch et al., 2012).

Corneal epithelium itself has further layering consisting of a basal layer of newly formed columnar cells; these are pushed progressively towards the surface as new cells develop; at this stage they are polyhedral shaped. Finally there is the superficial layer of polygonal shaped squamous cells, these cells have microvilli to aid mucus adhesion, which further aids adherence of the tear film. Superficial epithelial cells are surrounded by Ca^2+^ dependent cell binding sites, zonula occludens, otherwise known as tight junctions, immediately below these are zonula adherens, and towards the base of the cells there are spot like contact zones known as desmosomes. The combination of these cell membrane adherent regions and the lipophilicity of epithelia provide a very effective barrier function that protects the eye from ingress of alien material (Jain-Vakkalagadda et al., 2004; Kaur and Batra, 2007).

The effectiveness of the epithelium barrier is dependent on an undetermined concentration of Ca^2+^ ions being available to the plasma membrane. Calcium sequestering compounds are effective at disrupting corneal epithelia by extracting Ca^2+^ ions (Kaur and Smitha, 2002).

Polyaminocarboxylic acids are a class of synthetic compounds with metal ion sequestering properties. Ethylenediaminetetraacetic acid (EDTA) is an effective calcium chelator which is included in many ocular drug formulations as a preservative and stabiliser (Freeman and Kahook, 2009; Grass et al., 1985); however there are concerns regarding its accumulative toxicity towards intraocular structures when used in the longer term. The present study investigates the use of EDTA together with two analogues, ethylenediamine-*N*,*N*′-disuccinic acid (EDDS) and ethylene glycol-bis(2-aminoethylether)-*N*,*N*,*N*′,*N*′-tetraacetic acid (EGTA) for their effectiveness as penetration enhancers in delivery of riboflavin for the treatment of keratoconus and other corneal disorders (Fang-Sheng et al., 1992; Grass et al., 1985; Meers et al., 2005; Oviedo and Rodriguez, 2003). Around 1 in 2000 of the general population is afflicted with keratoconus, a debilitating ocular condition whereby the cornea degenerates, and the patient’s vision becomes seriously compromised. The condition can develop to the stage where penetrating keratoplasty (corneal transplant) becomes necessary (Tuft et al., 1994). Wollensak et al. (2003) published their work on development of a novel procedure for treating keratoconus by ultraviolet A induced riboflavin–collagen cross-linking. This procedure has now become a common practice for this and other corneal degenerative conditions. However, the procedure relies on physical abrasion of the epithelium under anaesthesia to allow corneal permeation of riboflavin solution into the stroma. Rupenthal et al. (2011) report on what they term ‘the corneal wound healing cascade’ initiated when corneal epithelia sustain injury, cytokines are released leading to keratocyte apoptosis followed by several more events before eventual return to normality. Development of a means to deliver this drug without resorting to epithelial debridement would be a major improvement on the procedure.

The present study investigates the effects of EDTA, EGTA and EDDS calcium sequestering compounds on corneal permeability of riboflavin after first determining their Ca^2+^ binding ability using isothermal titration calorimetry (ITC). We also explored corneal integrity after exposure to these solutions. Analysis of these solutions before and after corneal exposure was carried out using atomic absorption spectrometry (AAS), and it was established that they are able to extract calcium ions from corneal epithelial membranes. Corneal barrier function was evaluated using transepithelial electrical resistance analysis (TEER) to determine changes in electrical resistance of the cornea after exposure to Ca^2+^ chelating solutions. A reduction in electrical resistance correlates to increased permeability, supporting the hypothesis that tight junctions are dependent on Ca^2+^ availability (Kikuchi et al., 2005). Permeability studies using Franz diffusion cells (FDC) confirmed that polyaminocarboxylic acids enhance corneal permeability of riboflavin compared to the drug dissolved in PBS. Examination using microscopy has shown that the histology of the epithelial barrier was altered when they were exposed to these compounds.

## 2 Materials and methods

### 2.1 Materials

Ethylenediaminetetraacetic acid (EDTA), ethylenediamine-*N*,*N*′-disuccinic acid (EDDS), ethylene glycol-bis(2-aminoethylether)-*N*,*N*,*N*′,*N*′-tetraacetic acid (EGTA), 2-(*N*-morpholino) ethanesulfonic acid (MES), riboflavin, sodium hexane-1-sulfonate monohydrate and glacial acetic acid were purchased from Sigma–Aldrich (Gillingham, UK). 1000 ppm calcium calibration standard in nitric acid, 10% w/v lanthanum chloride, calcium chloride, sodium chloride, potassium chloride, sodium phosphate, potassium dihydrogen phosphate, sodium hydroxide, hydrochloric acid, optimal cutting temperature compound (OCT) and HPLC grade ethanol were obtained from Fischer Scientific (Hemel Hempstead, UK). Vectashield mounting medium with 4′,6-diamidino-2-phenylindole (DAPI) were obtained from Vector Laboratories Ltd. (Peterborough, UK). MilliQ ultrapure water (18 mΩ cm^−1^) was used for all aqueous solutions. All materials were used as supplied without modification.

### 2.2 Preparation of buffer solutions

Isotonic PBS was prepared in-house and was adjusted to pH 7.4 ± 0.2 using 0.1 M NaOH solution (Roskams and Rodgers, 2002). Ion-pair buffer for HPLC analysis was prepared using sodium hexane-1-sulfonate monohydrate (0.1 M) adjusted to pH 3.0 ± 0.2 using glacial acetic acid (Anyakora et al., 2008). For isothermal titration calorimetry, MES (10 mM) buffer was prepared and adjusted to pH 7.4 ± 0.2 using 1 M NaOH solution.

### 2.3 Isothermal titration calorimetry

ITC analysis was used to determine the Ca^2+^ binding properties of EDTA, EGTA and EDDS using Microcal model ITC200 calorimeter (cell volume = 200 μL), with ITC200 software version 1.24.2 (MicroCal Inc., USA). Data acquisition and graphical analysis was achieved using Origin 7 SR4, v7.0552 software (OriginLab Corporation, USA). Water was placed in the reference cell and 10 μCal s^−1^ selected for reference power. 0.4 mM solutions of EDTA, EGTA and EDDS in 10 mM MES buffer (pH 7.4 ± 0.2) were titrated by CaCl_2_ in 10 mM MES buffer (pH 7.4 ± 0.2). Calcium chloride titrant at 6 mM was used for EDTA and EGTA, a higher concentration at 25 mM was used for EDDS in order to reach saturation. A method with an initial injection of 0.4 μL followed by 15 injections of 2 μL with spacing of 150 s; analysis was carried out at 25 °C.

### 2.4 Atomic absorption spectroscopy (AAS)

Calcium ion analysis was conducted using a novAA 350 system with WinAAS software, version 4.5.0 (Analytik JENA, Germany). Following the method for calcium analysis published in ‘*Atomic Absorption Data Book*’, briefly, 50 mm stoichiometric air/acetylene flame, 422.7 nm wavelength, ultrapure water (18 mΩ cm^−1^) as the carrier (Whiteside and Milner, 1983). Quantitation achieved by reference to a calibration curve produced from Ca^2+^ standards in ultrapure water incorporating 5% w/v lanthanum chloride as a releasing agent. Standards were prepared at Ca^2+^ concentrations ranging from 0.1 to 10.0 ppm (*r*^2^ = 0.9921). Samples are diluted for analysis by a factor of 100, therefore a lower concentration of aqueous LaCl_3_ at 0.25% w/v is used for their preparation.

### 2.5 Transepithelial electrical resistance (TEER)

Adapted low volume (5 mL each side) Ussing chambers were used with an EVOM^2^ Epithelial Voltohmmeter and STX2 electrode modified by increasing the spacing between electrodes to fit the experiment cells (World Precision Instruments, Inc., USA). The Voltohmmeter was charged overnight prior to experiment and calibrated with the supplied 1000 Ω resister after 1 h equilibration. All solutions were degassed by sonication for 20 min, and experiments were carried out at 34 ± 1 °C to mimic the physiological temperature at the corneal surface (Fujishama et al., 1996; Liu et al., 2011). Determination of baseline electrical resistance of the PBS solution in the experiment cell was measured without a membrane in place preceding measurement of corneal resistance. An initial measurement was taken at <5 min (*t*_0_) then at 30 min intervals for 2 h. PBS solution (pH 7.4) was used in the receiving chamber, and solutions of 1 mg mL^−1^ EDTA, EGTA or EDDS in PBS were added to the donor chamber. TEER values were calculated using the following equation:

(1) $$\text{TEER}=\text{Resistance},\text{kΩ}\times \text{membrane}\text{area},{\text{cm}}^{2}$$

### 2.6 HPLC analysis

Analysis was conducted using a PerkinElmer series 200 HPLC system comprising of 785 A UV–vis detector, series 200 quaternary pump and series 200 autosampler (PerkinElmer Inc., UK), Hamilton PRP-1 reversed phase column, 150 mm × 4.1 mm, 10 μm (part number: 79425) and data acquisition software (Peaksimple, version 4.09, SRI Inc., USA). Analysis of riboflavin was achieved with a run time of 5 min using the method developed by us. Isocratic conditions were used at 25 °C with the mobile phase comprising of 20% ethanol and 80% ion-pair buffer, flow rate 0.8 mL min^−1^, 10 μL injection volume, UV detector at 267 nm corresponding to riboflavin absorption *λ*_max_, a retention time of 3.05 min. Quantitation was achieved by reference to a calibration curve produced from riboflavin standards in PBS, pH 7.4 ± 0.2 at concentrations ranging from 0.01 to 25 μg mL^−1^ (*r*^2^ = 0.9996).

### 2.7 Preparation of animal tissues

For Franz diffusion cell experiments, bovine corneas were dissected within 4 h of slaughter using a sharp blade, the cornea with 2–3 mm of sclera was carefully excised, rinsed with PBS, wrapped in cling film to prevent dehydration and stored for up to four days at 4 °C. For whole eye experiments, the eyes were stored for up to two days at 4 °C. Each experiment was carried out in triplicate using a different eye or cornea for each repeat.

### 2.8 Whole eye experiments

Using an experimental procedure previously developed and reported by us (Morrison et al., 2013), briefly, fresh whole bovine eyes were placed in 150 mL beakers, cornea facing uppermost. A Franz diffusion cell donor chamber was placed on the cornea as a means to contain the dose; the setup was wrapped in cling-film ensuring a good seal between the donor chamber and the cornea; these were placed in a water bath at 34 ± 1 °C. Individual eyes were exposed to PBS or solutions of EDTA, EGTA or EDDS at 1 mg mL^−1^ in PBS, all at pH 7.4 ± 0.2 by placing 1 mL solution onto the cornea. After 3 h, the solutions were recovered and placed in micro-centrifuge tubes, centrifuged at 10,000 rpm for 10 min. Samples of solutions before corneal exposure and each supernatant from cornea exposed solutions were diluted by 100 in 0.25% w/v LaCl_3_ aqueous solution for analysis using established methods for atomic absorption spectrometry.

### 2.9 Corneal integrity

From the above experiment, corneas were dissected after exposure, and sections were prepared for microscopy by setting cornea segments in OCT, quick freezing on dry ice and subsequent sectioning. Specimens of 7 μm thickness were prepared using a microtome (Bright, model 5040, UK) and cryostat (Bright, model OTF, UK) placed in groups of four to six on 75 mm × 25 mm glass slides, dried in a warm air stream for ∼10 min. Specimens were stained using Vectashield with DAPI mounting medium, and a glass coverslip applied. All cornea sections were examined within seven days of preparation using an AXIOCAM MRm 1.3 MP digital camera attached to a Zeiss AXIO Imager A1 fluorescent microscope, using AXIO Vs 40 V.4.8.2.0 software (Zeiss, Oberkochen, Germany). A 10× magnification eyepiece together with a 5× magnification objective lens was employed, and DAPI fluorescence filter was selected.

### 2.10 Effect of calcium sequestering compounds on riboflavin permeability

Corneal permeability of riboflavin was studied *in vitro* using Franz diffusion cells. Solutions of EDTA, EDDS and EGTA at concentration of 1 mg mL^−1^ in PBS, pH 7.4 ± 0.2 were prepared; riboflavin was added at 0.1 mg mL^−1^. A solution of riboflavin in PBS, pH 7.4 ± 0.2 was also prepared as a control. Bovine corneas were mounted in Franz diffusion cells (FDC), epithelium side facing the donor compartment. The receiver compartment was filled with pre-warmed and degassed PBS (16.5 mL). Prepared FDCs were placed in a water bath, stirred at 34 ± 1 °C. 1 mL of the prepared solutions were added to the donor compartment and sealed with film to prevent evaporation. Riboflavin is known to be photodegradable (Terekhova et al., 2011), therefore the whole experiment was shielded with aluminium foil. Sink conditions were employed with 0.4 mL aliquots taken every 60 min for 240 min. HPLC analysis was carried out immediately after all samples had been collected to avoid drug degradation. Apparent permeability and steady-state flux were calculated using the following equation:

(2) $$P_{\text{app}}=\frac{\text{Δ}Q}{\text{Δ}t60AC_{0}}$$

where *P*_app_ is the apparent permeability of riboflavin through bovine cornea, *Q* is total drug permeated at time *t*, Δ*Q*/Δ*t* is the steady-state flux into the receiving solution (μmol min^−1^) which equates to the gradient of the linear portion of the graph, 60 is minutes to seconds conversion factor, *A* is the area of exposed cornea (1.54 cm^2^) and *C*_0_ is the initial amount of drug added to the donor chamber (Jarho et al., 1996).

### 2.11 Cornea extracts

Corneas from the riboflavin permeability experiment were trimmed of sclera, gently blotted with tissue paper, weighed and placed in glass vials to which 5 mL absolute ethanol was added to extract absorbed riboflavin. Differences in its accumulation within the corneas between different solutions were compared. The vials were stored at room temperature shielded from light for three days. From these vials, 1 mL of extract solvent was centrifuged at 10,000 rpm for 10 min, and the supernatant transferred to autosample vials for HPLC analysis.

## 3 Results and discussion

### 3.1 Ca^2+^ sequestering properties of EDTA, EGTA and EDDS

EDTA is already known for its Ca^2+^ sequestering properties and has applications in many industries including cosmetics, food production and pharmaceuticals (Oviedo and Rodriguez, 2003). EDDS and EGTA are analogues, and these were investigated as possible alternatives to potentially enhance the permeability of riboflavin into the cornea. Ca^2+^ plays an important role in the barrier function of corneal epithelium, and it was hypothesised that extracting these ions from the corneal membrane could enhance drug permeation. Ca^2+^ binding efficiency was investigated using isothermal titration calorimetry. Atomic absorption spectrometry was used to study Ca^2+^ extraction from the cornea, and corneal integrity was determined by examination using fluorescence microscopy. *In vitro* studies compared riboflavin permeability enhancement through the cornea, and solvent extraction was used to determine the levels of riboflavin taken up by the cornea. TEER analysis measured changes in electrical resistance of the cornea, and this correlates with changes in riboflavin permeability.

### 3.2 Isothermal titration calorimetry

**Fig. 1:**
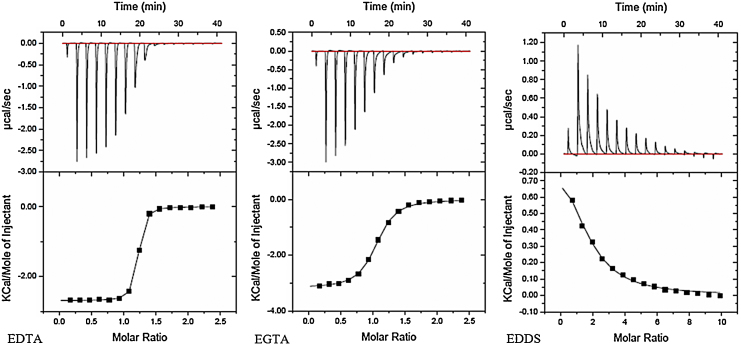
Isotherms for Ca^2+^ titration of 0.4 mM EDTA, EGTA and EDDS, concentration of Ca^2+^ was 6 mM for EDTA and EGTA, and 25 mM for EDDS.

ITC was used to determine Ca^2+^ binding efficiency of EDTA, EGTA and EDDS solutions at physiological pH 7.4. Fig. 1 shows individual raw data and corresponding isotherms of the respective compounds titrated with CaCl_2_. Traces for EDTA and EGTA show an exothermic trend, which agrees with the literature (Griko, 1999; Keowmaneechai and McClements, 2002; Henzl et al., 2003), whilst the interaction of EDDS with Ca^2+^ is endothermic under the experimental conditions. Measured values of enthalpy (Δ*H*), stoichimetry (*N*), binding affinity (*K*_b_) and entropy (Δ*S*) from this analysis are shown in Table 1. EDTA and EGTA have strong binding affinity (*K*_b_) of (1.20 ± 0.17) × 10^6^ M^−1^ and (1.41 ± 0.26) × 10^5^ M^−1^, respectively, whereas EDDS has a much lower *K*_b_ of (3.06 ± 0.24) × 10^3^ M^−1^.

**Fig. 2:**
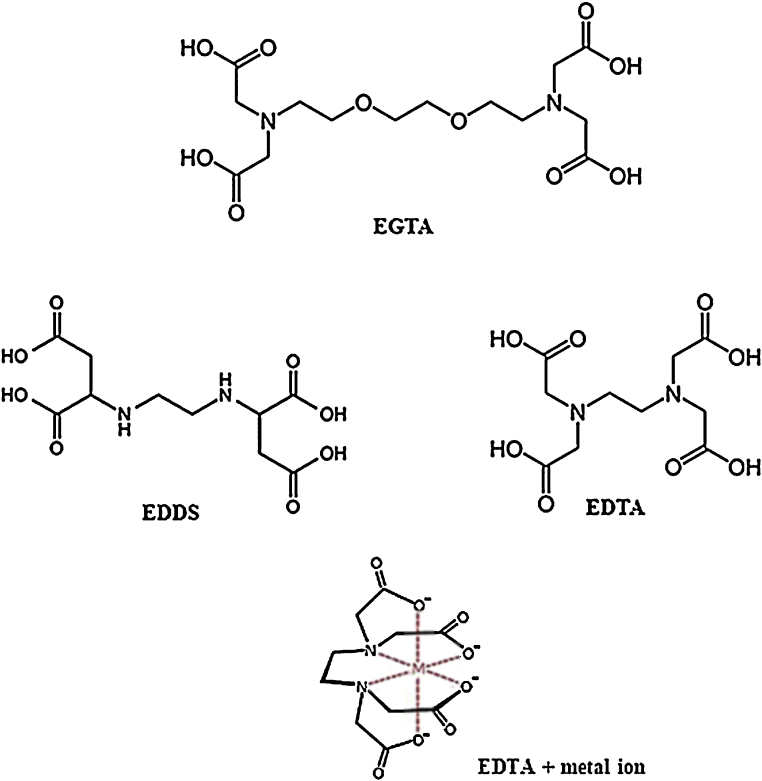
Chemical structures of EGTA, EDTA and EDDS and an example of EDTA/metal ion complex.

Complexation mechanisms of metal ion – chelator binding are not fully understood; however, we found that all 3 compounds investigated in this study have affinity for Ca^2+^ sequestration. Polyaminocarboxylic acids have pH dependent zwitterionic structures, *i.e.* they are molecules carrying multiple positive and negative charges. Metal ion affinity is more favourable under mildly acidic conditions for EDTA and possibly EGTA, whilst slightly alkaline conditions are required by EDDS (Yip et al., 2010). Hydrogen bonding and proton transfer play an important role in chemical complexation (Chandra et al., 2000; Ho et al., 2003). Fig. 2 shows the chemical structures of these 3 compounds and an example of metal ion complex (Zeng et al., 2014). They have different numbers of H-bond donor/acceptor sites: EDTA has 4 donor and 10 acceptor sites, EGTA has 4 donors and 12 acceptors, and EDDS has 6 donors and 10 acceptors. Secondly, the carboxylic acid structures of EDTA and EGTA are similar in that these groups are ‘open’ in comparison with the corresponding groups of EDDS where they form ring-like entities; in this case, there is more likelihood that the OH groups may form intramolecular H-bonds rather than interacting with Ca^2+^. The carbon chain link between the carboxylic acid groups at each side of the molecule is shorter for EDTA than for EGTA, therefore the distance for bonding between Ca^2+^ and carboxylic acid groups is less; this could be the reason for the order of magnitude difference in binding affinity between EDTA and EGTA. Considering these variations could explain the observed differences in Ca^2+^ sequestering performance between these compounds.

### 3.3 Whole eye experiments

**Fig. 3:**
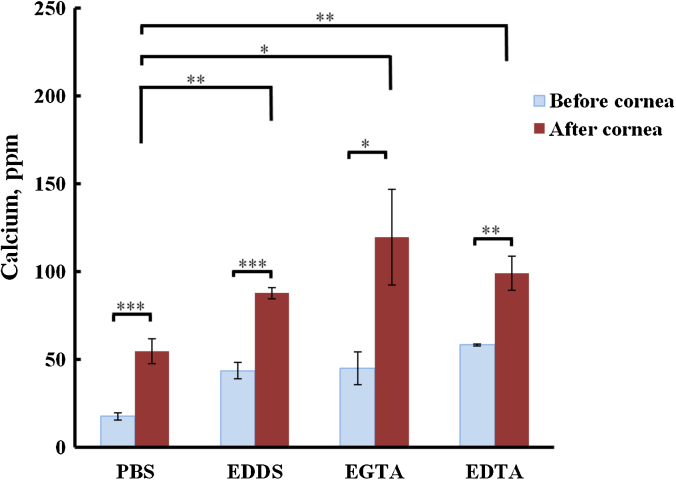
Calcium concentration in solutions containing PBS and Ca^2+^ sequestering compounds (1 mg mL^−1^) before and after 3 h exposure to bovine cornea. *P < 0.05, **P < 0.01, ***P < 0.001, one-way ANOVA, *n* = 3.

Experiments were conducted to determine Ca^2+^ extraction from the cornea using Ca^2+^ chelators. An area of cornea (1.54 ± 0.22 cm^2^) was exposed to calcium sequestering solutions at a concentration of 1 mg mL^−1^ in isotonic PBS at pH 7.4 ± 0.2, another set of eyes were exposed to PBS under the same conditions. After 3 h, the solutions were recovered, centrifuged at 10,000 rpm for 10 min and a sample of supernatant diluted with 0.25% w/v LaCl_3_ in ultrapure water (18 mΩ cm^−1^) for AAS analysis. Ca^2+^ is a persistent species within our environment, in water, on glassware and as an impurity in many reagent grade chemicals, therefore ultrapure water (18 mΩ cm^−1^) was used to rinse the glassware and to make up the aqueous solutions for these experiments. There still remains an element of Ca^2+^ contamination from impurities in reagents and our working environment, therefore calcium was measured in solutions before and after they were exposed to corneas. This investigation has shown that calcium sequestering compounds capture Ca^2+^ from the cornea above this ‘background’ level. Fig. 3 shows differences in calcium content of solutions before corneal contact compared to the same solutions after exposure. All formulations show a net increase in Ca^2+^ concentration at the end of this experiment, and the rank order of calcium sequestering performance from bovine cornea compared to PBS solution is PBS (54.67 ± 7.04 ppm) < EDDS**(87.60 ± 3.30 ppm) < EDTA**(98.97 ± 9.86 ppm) < EGTA*(119.57 ± 27.28 ppm); however, when applying statistical analysis for performance between EDTA, EGTA and EDDS there are no significant differences. *P < 0.05, **P < 0.01, ***P < 0.001, one-way ANOVA, *n* = 3.

### 3.4 TEER analysis

Transepithelial electrical resistance (TEER) measurement is a technique useful in determining the barrier function of epithelial membranes. These membranes are polar and selective to ion-transport whilst they exclude macromolecules (Tang and Goodenough, 2003; Brown and Stow, 1996). TEER values of membranes determine the effectiveness of their barrier function; higher values correspond to less permeable membranes and *vice versa*, therefore having a means to measure changes in resistance is a convenient tool that can be used to evaluate the effect of compounds applied to tissue.

**Fig. 4:**
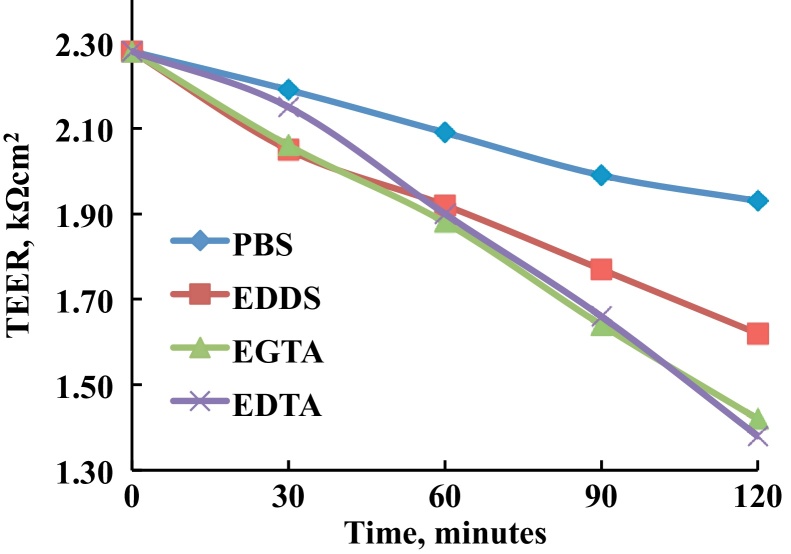
TEER analysis for PBS, EDDS, EGTA and EDTA exposed bovine corneas. Error bars have been omitted for clarity and are included on individual graphs in the supplementary information.

Baseline resistance of PBS solution in the experiment cells without a membrane was found to be 0.227 ± 0.01 kΩ, and this was deducted from the measured corneal TEER values. Measurements for corneal resistance using PBS (control) and EDDS, EGTA and EDTA solutions (1 mg mL^−1^) in the donor chambers were taken. Fig. 4 shows the differences in calculated TEER values at 30 min intervals up to 120 min after deducting the baseline between the control and chelator exposed corneas. The initial TEER values recorded for bovine corneas were 2.28 ± 0.06 kΩ cm^2^, which is in good agreement with the literature (Kikuchi et al., 2005). Electrical resistance reduced for corneas exposed to PBS from 2.28 ± 0.06 to 1.93 ± 0.04 kΩ cm^2^ over 2 h, and this is due to the corneal swelling observed when using *in vitro* techniques. However, the reduction in electrical resistance was enhanced when using chelators in PBS. The values for these formulations were reduced to 1.62 ± 0.04, 1.42 ± 0.02 and 1.38 ± 0.03 kΩ cm^2^ over the same timescale for EDDS, EGTA and EDTA, respectively. At 120 min, TEER values were significantly reduced for PBS solutions containing 1 mg mL^−1^ of EDDS, EGTA or EDTA compared with the PBS control (one-way ANOVA, *P* < 0.01, *n* = 4). EGTA and EDTA show a significantly better ability to reduce TEER values compared to EDDS (one-way ANOVA, *P* < 0.01, *n* = 4).

### 3.5 Corneal integrity

**Fig. 5:**
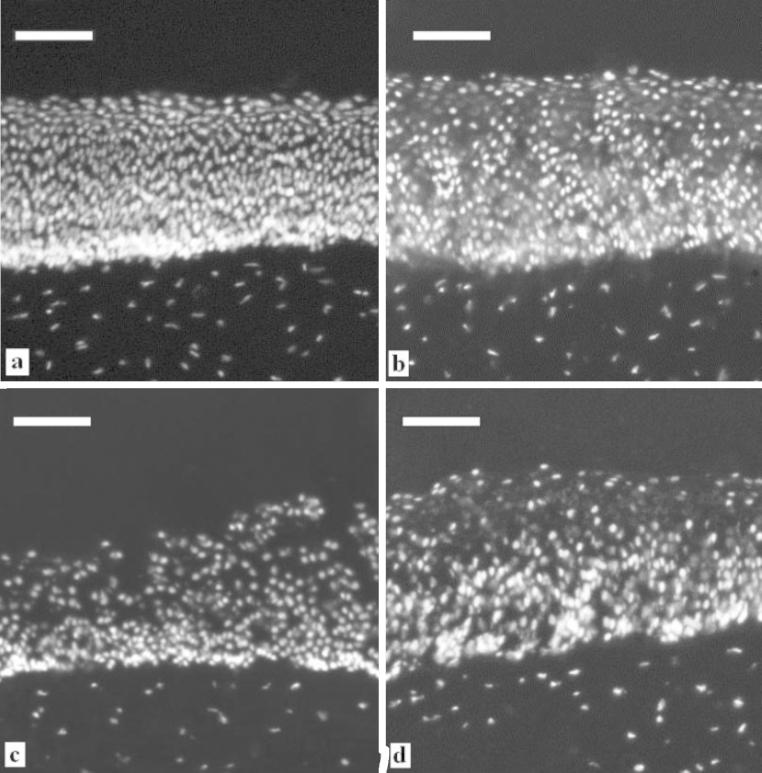
Micrographs showing histological changes due to exposure to calcium sequestering formulations at a concentration of 1 mg mL^−1^ for 3 h (b–d) compared to exposure to PBS (a). (a) PBS, (b) EDDS, (c) EGTA and (d) EDTA. Scale bar = 100 μm.

Previously we have reported a new technique to study the histological changes taking place in bovine cornea after exposing them to cyclodextrins (Morrison et al., 2013). In the present study we employed a similar method to investigate the effect of calcium chelators on the cornea. The dissected corneas from the whole eye experiment were examined using fluorescence microscopy, and there is clear evidence of histological changes between corneas exposed PBS or Ca^2+^ sequestering formulations in PBS (Fig. 5). The cornea shown in Fig. 5a was exposed to PBS, Fig. 5b EDDS, Fig. 5c EGTA and Fig. 5d EDTA, all at pH 7.4 ± 0.2 with calcium sequestering formulations at 1 mg mL^−1^ in PBS. The epithelium shows no evidence of disruption for the corneas exposed to PBS (Fig. 5a), whilst the solutions of PBS with calcium chelators show disruption to the epithelium (Fig. 5b–d). EDDS and EDTA appear to cause some swelling with voids throughout this layer; perforation seems to penetrate the whole of the epithelium for EDTA. With EGTA, there is evidence of intense disruption to the superficial epithelial layers and a relatively undisturbed basal layer. These findings correlate with the above-discussed results where it was found that PBS had only moderate ability to extract Ca^2+^ upon corneal exposure, whereas the sequestering compounds were able to extract significantly more. EDDS extracted the least amount of Ca^2+^ closely followed by EDTA, whilst EGTA sequestered the largest amount.

### 3.6 Corneal permeability of riboflavin

**Fig. 6:**
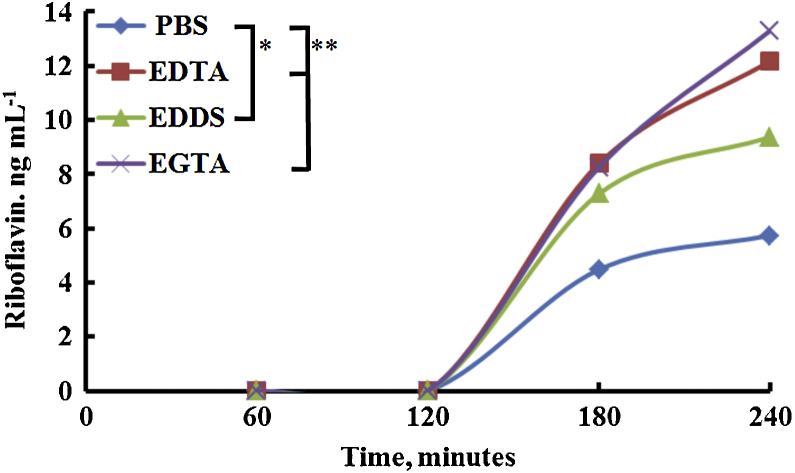
Calcium chelator enhanced riboflavin permeability through bovine cornea (chelators at 1 mg mL^−1^ with riboflavin at 0.1 mg mL^−1^). Error bars have been omitted for clarity and have been included on individual plots in supporting information. *P < 0.05, **P < 0.01, one-way ANOVA, *n* = 4.

This part of the investigation compared the *in vitro* permeability of riboflavin at a concentration of 0.1 mg mL^−1^ in PBS (pH 7.4 ± 0.2) to the same concentration of riboflavin in PBS with EDTA, EDDS or EGTA at 1 mg mL^−1^ (pH 7.4 ± 0.2) through bovine cornea using Franz diffusion cells. For all solutions, a lag-time of 120 min was observed before any evidence of riboflavin permeation was detected, after which any differences in riboflavin permeability through the cornea between the various formulations could be measured (Fig. 6); this is in good agreement with our results on Ca^2+^ extraction, TEER analysis and corneal integrity. The factors affecting the observed lag time are due the initial accumulation of riboflavin into the stromal reservoir until there is sufficient amount to further transport out from the cornea into the receiving solution. Once riboflavin starts to move through the cornea, its concentration within the receiving solution would initially be below the limit of quantification which is ∼1 ppm, increasing with time. At 240 min the measured concentration of riboflavin in the receiving solution was 5.74 ± 0.96 ng mL^−1^ in PBS, 9.38 ± 3.89 ng mL^−1^ in EDDS, 12.18 ± 2.76 ng mL^−1^ in EDTA, and 13.30 ± 1.40 ng mL^−1^ in EGTA.

Calculating the steady state flux and apparent permeability (*P*_app_) of riboflavin across bovine cornea was shown to be enhanced using calcium sequestering formulations compared to riboflavin in PBS. EGTA performed the best, closely followed by EDTA then EDDS with *P*_app_ of 4.508 × 10^−3^, 4.130 × 10^−3^ and 3.182 × 10^−3^ cm s^−1^, respectively, this compares with riboflavin in PBS at 1.945 × 10^−3^ cm s^−1^ (Table 2).

### 3.7 Cornea extracts

**Fig. 7:**
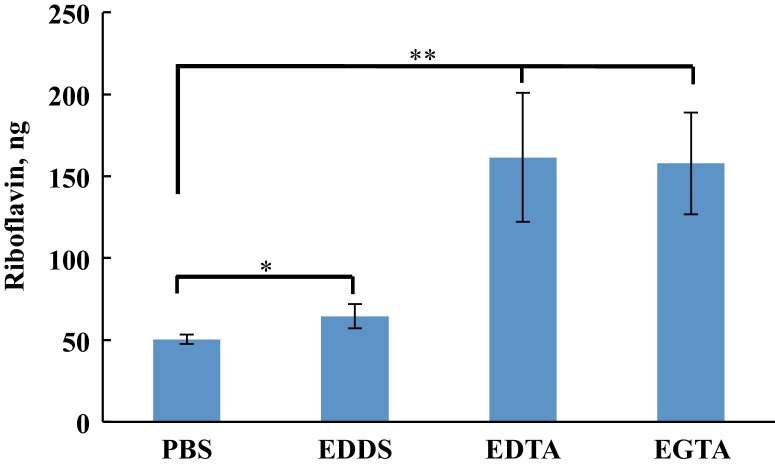
Comparative enhancement of riboflavin absorption into bovine corneas using calcium chelators (chelators at 1 mg mL^−1^ with riboflavin at 0.1 mg mL^−1^). *P < 0.05, **P < 0.01, one-way ANOVA, *n* = 3.

When treating keratoconus, the aim is to deliver riboflavin into the corneal stroma where it then mediates UVA induced collagen cross-linking, therefore for this part of the study the amount of drug absorbed into the cornea for each different formulation was measured. During the riboflavin permeability experiments above, corneas were dosed with riboflavin formulations in PBS with or without calcium sequestering compounds. At the end of the riboflavin permeability experiments, the FDC’s were disassembled, and the corneas were trimmed to exclude any scleral tissue. Riboflavin was extracted using ethanol with subsequent analysis by HPLC. Fig. 7 shows the differences in amount of riboflavin absorbed by corneas using different formulations; there is a significant difference between experiments for riboflavin in PBS and formulations which include calcium chelators. In the corneas where riboflavin in PBS was applied, we see a mean amount of 50.41 ± 2.80 ng riboflavin extracted, formulations including EDDS show a slight but significant improvement at 64.41 ± 7.41 ng, for EDTA and EGTA we see a marked increase to 161.48 ± 39.34 ng and 157.75 ± 31.00 ng, respectively.

Table 3 shows the calculated values of extracted riboflavin in picogram per milligram of cornea. Comparing these values with those for riboflavin in PBS which had a value of 62.46 pg mg^−1^, EDDS had a slightly higher but significant mean riboflavin content of 76.08 pg mg^−1^, EDTA had a much higher value of 180.44 pg mg^−1^ and EGTA had the highest level at 187.54 pg mg^−1^.

These results correlate with the findings from other experiments within this investigation.

## 4 Conclusions

In this series of experiments we tested the hypothesis that calcium plays an important role in the barrier function of the cornea against riboflavin penetration. Ca^2+^ sequestration is possible using polyaminocarboxylic acids, and these compounds were central to this investigation. The well-known and reported compound EDTA was compared with 2 of its analogues, EGTA and EDDS and their performance against a PBS control was compared from many angles. First, their ability to bind with Ca^2+^ was measured using isothermal titration calorimetry, and it was found that as compared with PBS polyaminocarboxylates have superior capacity to form complexes with calcium ions. Second, using atomic absorption spectrometry, their ability to extract Ca^2+^ from the corneal epithelium was shown to be significantly higher than solutions without these compounds when formulations were analysed before and after corneal exposure. Transepithelial electrical resistance was measured for corneas exposed to these compounds and compared to TEER values of corneas exposed to PBS. It was demonstrated that Ca^2+^ chelators were effective at reducing electrical resistance of corneal membranes, supporting the hypothesis that Ca^2+^ plays an important role in maintaining epithelial barrier function. Next, any effect on corneal integrity was examined using microscopy, and some very distinct histological changes became evident between the PBS control and sequestering formulations. Crucially, the next set of experiments tested any enhancement to riboflavin permeability through the cornea, and it was found that all the sequestering compounds investigated in this study brought improved drug permeability compared with riboflavin in PBS. Finally the amount of riboflavin absorbed by the cornea was determined in an experiment designed to extract the drug from corneas used in these experiments, and again it was found that formulations incorporating polyaminocarboxylates were superior to formulations without any enhancement. Across the whole series of experiments, a pattern emerged; it was found that all Ca^2+^ sequestering formulations performed better than the PBS control. In all cases, EDDS brought moderate enhancement, whilst EDTA and EGTA brought a marked improvement in performance. Further work could consider implications regarding epithelial recovery after treatment using calcium chelators.

## Figures and Tables

**Table 1:** ITC analysis at pH 7.4 ± 0.2 of EDTA, EGTA and EDTA titrated with CaCl_2_.

Chelator in 10 mM MES	CaCl_2_ in 10 mM MES	Stoichiometry, *N*	Binding affinity, *K*_b_ (M^−1^)	Δ*H* (kJ mol^−1^)	Δ*S* (kJ mol^−1^ deg^−1^)
EDTA (0.4 mM)	6 mM	1.12 ± 0.06	(1.20 ± 0.17) × 10^6^	−11.55 ± 0.25	77.70 ± 1.88
EGTA (0.4 mM)	6 mM	1.34 ± 0.29	(1.41 ± 0.26) × 10^5^	−13.10 ± 0.42	54.68 ± 2.80
EDDS (0.4 mM)	25 mM	1.55 ± 0.04	(3.06 ± 0.24) × 10^3^	4.03 ± 0.38	80.21 ± 0.63

**Table 2:** Steady state flux and apparent permeability of riboflavin in PBS with and without calcium sequestering compounds.

Riboflavin solution (0.266 μM) in:	Steady-state flux (μmol min^−1^)	*P*_app_ (cm s^−1^ × 10^−3^)
PBS	0.0478 ± 0.0080	1.945 ± 0.325
EDTA (1 mg mL^−1^)	0.1015 ± 0.0230	4.130 ± 0.935
EDDS (1 mg mL^−1^)	0.0782 ± 0.0324	3.182 ± 1.319
EGTA (1 mg mL^−1^)	0.1108 ± 0.0117	4.508 ± 0.475

**Table 3:** Riboflavin extracted from treated corneas, pg:mg.

Solution (pH 7.4 ± 0.2)	Riboflavin pg mg^−1^ cornea
PBS	62.46 ± 0.98
EDDS (1 mg mL^−1^)	76.08 ± 7.46**
EDTA (1 mg mL^−1^)	180.44 ± 38.55**
EGTA (1 mg mL^−1^)	187.54 ± 30.16**

** *P* < 0.01, one-way ANOVA, *n* = 3.
