# Supplementary material for: Enhancement in corneal permeability of riboflavin using calcium sequestering compounds
Source: Int J Pharm. 2014 Sep 10;472(1-2):56–64. doi: 10.1016/j.ijpharm.2014.06.007 (PMC4111866; doi:10.1016/j.ijpharm.2014.06.007)
Supplement: Supplementary file 1 [file mmc1.docx]

# Supplementary information to

Enhancement in Corneal Permeability of Riboflavin Using Calcium Sequestering Compounds

Peter W. J. Morrison, Vitaliy V. Khutoryanskiy*

School of Pharmacy, University of Reading, Whiteknights, PO Box 224, Reading, RG6 6AD, United Kingdom.

**Figure S1.** TEER for PBS exposed bovine cornea with error bars. Mean (n=3) ± standard deviation.

**Figure S2.** TEER for EDDS (aqueous, 1 mg mL^-1^) exposed bovine cornea with error bars. Mean (n=3) ± standard deviation.

**Figure S3.** TEER for EGTA (aqueous, 1 mg mL^-1^) exposed bovine cornea with error bars. Mean (n=3) ± standard deviation.

**Figure S4.** TEER for EDTA (aqueous, 1 mg mL^-1^) exposed bovine cornea with error bars. Mean (n=3) ± standard deviation.

**Figure S5.** Riboflavin (0.1 mg mL^-1^ in PBS, pH 7.4) permeability through bovine cornea with error bars. Mean (n=3) ± standard deviation.

**Figure S6.** Riboflavin (0.1 mg mL^-1^ in PBS, with EDDS at 1 mg mL^-1^, pH 7.4) permeability through bovine cornea with error bars. Mean (n=3) ± standard deviation.

**Figure S7.** Riboflavin (0.1 mg mL^-1^ in PBS, with EDTA at 1 mg mL^-1^, pH 7.4) permeability through bovine cornea with error bars. Mean (n=3) ± standard deviation.

**Figure S8.** Riboflavin (0.1 mg mL^-1^ in PBS, with EGTA at 1 mg mL^-1^, pH 7.4) permeability through bovine cornea with error bars. Mean (n=3) ± standard deviation.
